# Renal arterial resistive index is associated with severe histological changes and poor renal outcome during chronic kidney disease

**DOI:** 10.1186/1471-2369-13-139

**Published:** 2012-10-25

**Authors:** Naïke Bigé, Pierre Patrick Lévy, Patrice Callard, Jean-Manuel Faintuch, Valérie Chigot, Virginie Jousselin, Pierre Ronco, Jean-Jacques Boffa

**Affiliations:** 1 Department of Nephrology, AP-HP, Hôpital Tenon, 4 rue de la Chine, Paris, F-75020, France; 2 Public Health Department, AP-HP, Hôpital Tenon, Paris, F-75020, France; 3 INSERM UNIT 707, Paris, F-75012, France; 4 Université Pierre et Marie Curie-Paris 6, UMR-S 707, Paris, F-75012, France; 5 Department of Pathology, AP-HP, Hôpital Tenon, Paris, F-75020, France; 6 Department of Radiology, AP-HP, Hôpital Tenon, Paris, F-75020, France; 7 INSERM UNIT 702, Paris, F-75020, France; 8 Université Pierre et Marie Curie-Paris 6, UMR S 702 , Paris, France

**Keywords:** Chronic kidney disease, Arteriosclerosis, Fibrosis, Renal doppler

## Abstract

**Background:**

Chronic kidney disease (CKD) is a growing public health problem and end stage renal disease (ESRD) represents a large human and economic burden. It is important to identify patients at high risk of ESRD. In order to determine whether renal Doppler resistive index (RI) may discriminate those patients, we analyzed whether RI was associated with identified prognosis factors of CKD, in particular histological findings, and with renal outcome.

**Methods:**

RI was measured in the 48 hours before renal biopsy in 58 CKD patients. Clinical and biological data were collected prospectively at inclusion. Arteriosclerosis, interstitial fibrosis and glomerulosclerosis were quantitatively assessed on renal biopsy in a blinded fashion. MDRD eGFR at 18 months was collected for 35 (60%) patients. Renal function decline was defined as a decrease in eGFR from baseline of at least 5 mL/min/ 1.73 m^2^/year or need for chronic renal replacement therapy. Pearson’s correlation, Mann–Whitney and Chi-square tests were used for analysis of quantitative and qualitative variables respectively. Kaplan Meier analysis was realized to determine renal survival according to RI value using the log-rank test. Multiple logistic regression was performed including variables with p < 0.20 in univariate analysis.

**Results:**

Most patients had glomerulonephritis (82%). Median age was 46 years [21–87], eGFR 59 mL/min/ 1.73m^2^ [5–130], percentage of interstitial fibrosis 10% [0–90], glomerulosclerosis 13% [0–96] and RI 0.63 [0.31-1.00]. RI increased with age (r = 0.435, p = 0.0063), pulse pressure (r = 0.303, p = 0.022), renal atrophy (r = −0.275, p = 0.038) and renal dysfunction (r = −0.402, p = 0.0018). Patients with arterial intima/media ratio ≥ 1 (p = 0.032), interstitial fibrosis > 20% (p = 0.014) and renal function decline (p = 0.0023) had higher RI. Patients with baseline RI ≥ 0.65 had a poorer renal outcome than those with baseline RI < 0.65 (p = 0.0005). In multiple logistic regression, RI≥0.65 was associated with accelerated renal function decline independently of baseline eGFR and proteinuria/creatininuria ratio (OR=13.04 [1.984-85.727], p = 0.0075). Sensitivity, specificity, predictive positive and predictive negative values of RI ≥ 0.65 for renal function decline at 18 months were respectively 77%, 86%, 71% and 82%.

**Conclusions:**

Our results suggest that RI ≥ 0.65 is associated with severe interstitial fibrosis and arteriosclerosis and renal function decline. Thus, RI may contribute to identify patients at high risk of ESRD who may benefit from nephroprotective treatments.

## Background

Chronic kidney diseases (CKD) represent a growing public health problem [1]. Only few patients will experienced rapid renal function decline [2] and fewer will reach end stage renal disease [3]. Prediction of renal function outcome is a critical issue. Predictive factors include arterial hypertension, proteinuria and baseline renal function. In addition, interstitial fibrosis closely correlates to renal function and long-term prognosis [4] but in most patients, renal histology assessment is not performed. We interested in renal arterial resistive index (RI) because it can be measured not invasively by Doppler analysis of intrarenal arterial blood flow velocities and because its prognosis value has been proven in various clinical settings. These include the therapeutic management of renal artery stenosis [5,6]. During renal transplantation, increase in RI early after surgery is a marker of tubular necrosis [7] and, later on, is predictive of long-term graft dysfunction [8].

However, the clinical interest of RI in the course of CKD is still unclear. Twenty years ago, Platt et al. showed that RI was significantly higher in nephropathies with tubulo-interstitial and/or vascular injury than in isolated glomerulopathies. Later on, four studies analyzed the correlation between RI and histological changes associated with the progression of CKD [9-12]. However, several points make these results questionable. First of all, pathological criteria were not clearly defined in two of these studies [10,11]. Moreover, although authors agreed that RI increased with tubulo-interstitial injury, three studies [10-12] did not distinguish chronic lesions, such as tubular atrophy and interstitial fibrosis, from interstitial oedema and cellular infiltration, which may result from acute injury. Three studies tested the correlation between RI and glomerulosclerosis and found conflicting results [9,10,12]. Only two groups examined simultaneously the association of RI with various lesions associated with CKD, i.e. tubulo-interstitial, glomerular and vascular lesions. Whereas the first study showed an association of RI with arteriosclerosis, but not with arteriolosclerosis [10], the most recent one found that vascular lesions were globally associated with RI without distinction between arteriolar and arterial lesions [9]. Furthermore, the most accurate threshold of RI in clinical practice is still debated [12,13]. In parallel, several studies reported the correlation of RI with renal outcome in CKD [9,11,13-17].

Despite these encouraging results, renal Doppler remains underemployed for the management of CKD in clinical practice. We conducted a prospective study in patients who underwent renal biopsy for diagnosis of CKD. Our primary goal was to assess the association between pathological lesions and RI. The second objective was to determine the relation of RI with renal function outcome. The last purpose was to establish the most relevant threshold of RI in clinical practice.

## Methods

### Ethics statement

The study was approved by the ethic committee CPP Ile-de France 5 and written informed consent was obtained from all participants.

### Patients

We carried out a prospective study from October 2006 to November 2007 in 58 consecutive patients referred to the Nephrology department of the Tenon Hospital in Paris, France, who underwent a diagnosis renal biopsy. Inclusion criteria were the following: i) existence of a chronic kidney disease according to the KDOQI definition [18], i.e. estimated glomerular filtration rate (eGFR) < 60 mL/min/ 1.73 m^2^ and/or albuminuria ii) presence of at least 5 glomeruli on the biopsy sample, iii) renal US Doppler performed within two days before renal biopsy with a standardized measurement of RI. Patients were excluded from the study if they had renal artery stenosis, acute cardiac failure or hepato-renal syndrome.

### Clinical and biological data

Clinical (age, sex, treatments and blood pressure) and biological data (serum creatinine, eGFR according to the modified MDRD formula and proteinuria/creatininuria ratio) were collected prospectively at inclusion. Biochemical parameters were all measured in the biochemistry laboratory of the hospital.

MDRD eGFR at 6, 12 and 18 months after renal biopsy was collected in 46 (79%), 43 (74%) and 35 (60%) patients, respectively. In cohort studies, slope of eGFR decline is less than -5 mL/min/ 1.73 m^2^ in the majority of CKD patients [2,19-23]. In order to be close to clinical practice, we chose to define renal function decline as a decrease in eGFR of at least 5 mL/min/ 1.73 m^2^/year from baseline or need for chronic renal replacement therapy (RRT).

### Ultrasonographic doppler examination

US Doppler examination was performed in a standardized fashion by one of the two well-trained ultrasonographers selected for the study, in fasted patients. A SIEMENS ELEGRA SS device and a 3.5 MHz probe were used. For each patient, the maximal length of both kidneys was measured and added to obtain combined renal length. Arterial velocity signals were obtained from segmental or interlobar arteries in one kidney. Three records were performed at superior, medium and inferior poles. RI was calculated according to the Pourcelot’s formula: [(peak systolic velocity- end diastolic velocity)/ peak systolic velocity]. The mean of the three poles measures was used as the reference value of RI for each patient. In 15 patients, RI was measured in both kidneys with a Pearson’s correlation coefficient > 0.99.

### Histological examination

For each patient, histological analysis was performed by a unique senior pathologist who was unaware of US Doppler results. Light microscopy examination was performed on samples stained by H & E, PAS, Jones and Masson’s trichrome. The whole cortex was analyzed on eight serial sections of each biopsy under 25 to 400 × magnification.

**Figure 1:**
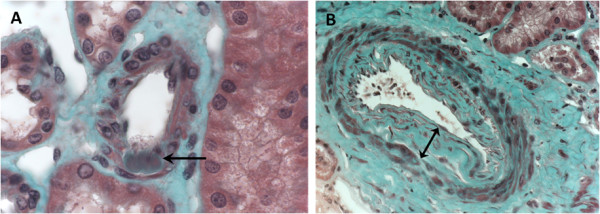
**Vascular lesions analyzed on renal biopsy (optic microscopy, Masson’s trichromic staining).A**: Arteriolosclerosis was defined as the presence of hyaline deposits (arrow) in the wall of at least one arteriole. **B**: Arteriosclerosis was defined as a thickening of intima. Maximal intima thickness visible on the sample biopsy was measured (double arrow). Here, intima/media ratio was superior to 1, which corresponds to severe arteriosclerosis.

Interstitial fibrosis was assessed visually as the percentage of fibrotic interstitial cortical tissue visible on Masson’s stain by 5%-stages [24]. Glomerulosclerosis was defined as the percentage of totally sclerotic glomeruli. Arteriolosclerosis was defined as the presence of hyaline deposits in the wall of at least one preglomerular arteriole (Figure 1A). Absence of arteriolosclerosis was asserted when none of the eight sections display hyaline deposits. Arteriosclerosis was defined as a thickening of the intima of at least one artery. Patients were classified in three groups according to the maximal intima thickness visible on the biopsy sample (Figure 1B): i) absence of arteriosclerosis: normal intima thickness, ii) moderate arteriosclerosis: thickening of intima with an intima/media ratio < 1, iii) severe arteriosclerosis: thickening of intima with an intima/media ratio ≥ 1.

### Statistical analysis

Number and percentage of patients, median and minimum-maximum values are provided for qualitative and quantitative values respectively. Linear relationship of RI with other variables was tested with Pearson’s correlation test. Analysis of parameters associated with RI and renal function decline were compared with Chi-square and Mann–Whitney tests respectively for qualitative and quantitative variables. Kaplan Meier analysis was realized to determine renal survival according to RI value using the log-rank test. In order to determine independent parameters associated with RI or renal function decline, we performed a multiple linear or logistic regression, respectively, including variables with p < 0.20 in univariate analysis.

Statistical analysis was performed using http://marne.u707.jussieu.fr/biostatgv/ website, GraphPad Prism 5.0 and StatView 5.0 softwares. A two-tailed p value < 0.05 was considered to be significant.

## Results

### Characteristics of patients

Fifty-eight patients were enrolled in the study according to the inclusion criteria. Their characteristics are summarized in Table 1. They were predominantly male (68.9%). Their median age was 49 years [23–89]. Their median eGFR and proteinuria/creatininuria ratio were respectively 59 mL/min/ 1.73 m^2^ [5–130] and 245 mg/mmol [7–2000]. Forty seven (81%) patients presented glomerular nephropathy. On biopsy, median sclerotic glomeruli and interstitial fibrosis percentage were respectively 13% [0–96] and 10% [0–90]. The presence of arteriolosclerosis or arteriosclerosis could not be studied in 5 and 12 patients, respectively, because of the absence of visible arteriole or artery on biopsy sample. Hyaline arterial deposits were observed on 25 of the 53 biopsies with visible arteriolar sections (47.2%). Arteriosclerosis was observed in 21 of the 46 patients (45.6%) for whom at least one artery was present on the biopsy. Eight (17.3%) patients had severe arteriosclerosis with an intima/media ratio ≥ 1 (Table 1). Median RI was 0.62 [0.31 – 1.0].

**Table 1:** Characteristics of patients at baseline

**Number of patients**	**58 (100%)**
**Age**	49 [23–89]
**Male**	49 (68.9%)
**Blood pressure (mmHg)**	
**Systolic**	130 (88–181)
**Diastolic**	78 (59–115)
**Pulse pressure**	50 (28–80)
**Renal function**	
** Serum creatinine (μmol/L)**	124 (54–906)
** eGFR (ml/min/1,73m**^**2**^**)**	59 (5–130)
** Proteinuria/creatininuria(mg/mmol)**	245 (7–2000)
**Antihypertensive treatments**	
** No antihypertensive drug**	27 (46.5%)
** 1 antihypertensive drug**	11 (19%)
** 2 antihypertensive drugs**	9 (15.5%)
** ≥ 3 antihypertensive drugs**	11 (19%)
** RAS blockers**	23 (39.6%)
**Renal biopsy**	
** % sclerotic glomeruli**	13 (0–96)
** % interstitial fibrosis**	10 (0–90)
** Vascular lesions**	34/51 (66.7%)
** Arteriolar hyaline deposits**	25/53 (47.2%)
** Intima/media ratio**	
** Normal**	25 (54.4%)
** < 1**	13 (28.3%)
** ≥ 1**	8 (17.3%)
**Diagnosis**	
** FSGN/HIVAN**	9 (15.5%)
** IgA nephropathy**	11 (19%)
** Membranous nephropathy**	7 (12.1%)
** Minimal change disease**	3 (5.2%)
** Lupus**	3 (5.2%)
** Vascularitis**	3 (5.2%)
** Membranous proliferative nephritis**	2 (3.4%)
** Diabetes**	2 (3.4%)
** Amyloidosis**	2 (3.4%)
** Other glomerulonephritis**	5 (8.6%)
** Acute tubulo-interstitial nephritis**	3 (1.7%)
** Chronic tubulo-interstitial nephritis**	2 (3.4%)
** Vascular nephropathy**	2 (3.4%)
** Other**	4 (6.9%)
**Renal arterial resistive index**	0.62 (0.31-1.00)

### Clinical and biological parameters associated with renal arterial resistive index

We found a positive correlation between RI and age (r = 0.435, p = 0.0063), pulse pressure (r = 0.303, p = 0.022) which are both associated with elevated arterial stiffness. Furthermore, RI was inversely correlated with baseline eGFR (r = −0.402, p = 0.0018) and to a lesser extent with combined renal length (r = −0.275, p = 0.038). In a multiple linear regression analysis including age, baseline eGFR, pulse pressure and combined renal length, only age (p = 0.0052) and baseline eGFR (p = 0.015) were independently associated with RI. We did not find any significant correlation of RI with proteinuria/creatininuria ratio (r = 0.141, p = 0.29), systolic (r = 0.080, p = 0.55) and diastolic blood pressure (r = −0.169, p = 0.21). RI was not different between patients who received an antihypertensive treatment and those who did not (p = 0.89). In the same way, RI did not differ between patients treated with renin angiotensin system (RAS) blockers (p = 0.65) and those who were not. RI was not influenced by the number of antihypertensive drugs prescribed (p = 0.87).

### Histological parameters associated with renal arterial resistive index

**Figure 2:**
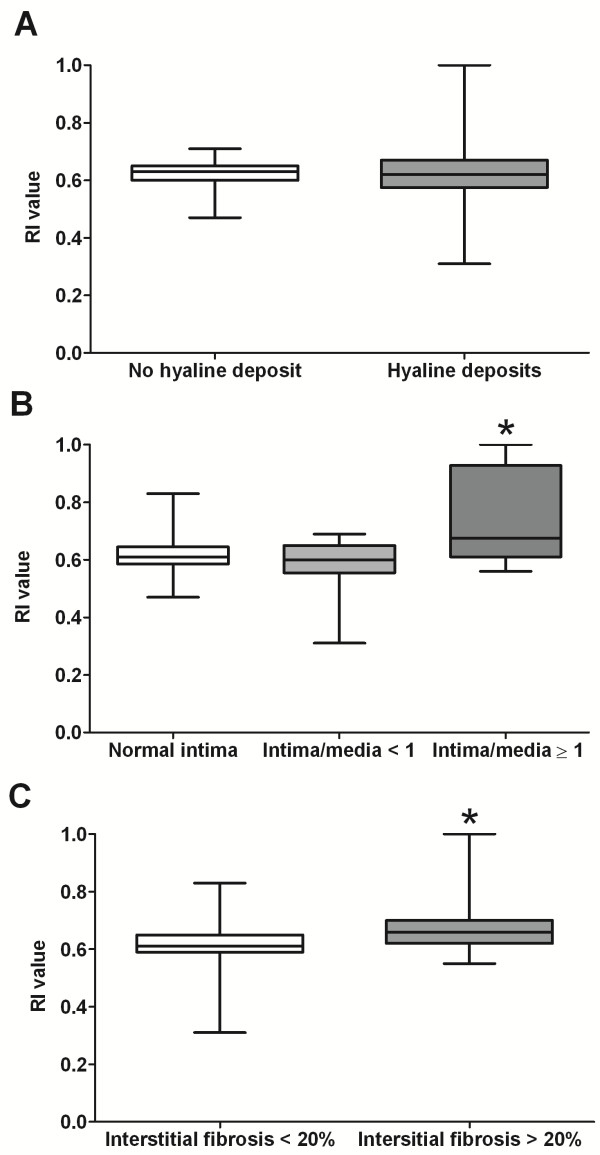
**Association of RI with histological parameters.** Boxes show the first and third quartiles, with the median as a thick line. Whiskers extend to minimum and maximum values. **A**: RI according to arteriolosclerosis, defined as the presence of hyaline deposits in the wall of at least one preglomerular arteriole. **B**: RI according to maximal intima thickness (moderate arteriosclerosis was defined as a thickening of intima with intima/media ratio < 1 and severe arteriosclerosis as an intima/media ratio ≥ 1). * p<0.05 versus normal and intima/media<1. **C**: RI according to interstitial fibrosis. * p<0.05 versus interstitial fibrosis<20%.

RI was not different whether hyaline deposits were present or not (0.62 [0.31-1.00] vs 0.63 [0.50-0.71], p = 0.99) (Figure 2A). RI value was similar in patients with normal intima or moderate intima thickening (intima/media ratio < 1) (0.62 [0.52-0.83] vs 0.60 [0.31-0.69], p = 0.71). In contrast, patients with severe arteriosclerosis (intima/media ratio ≥ 1) had a significantly higher RI than those with no or moderate arteriosclerosis (0.73 [0.56-1.00] vs 0.61 [0.31-0.83], p = 0.032) (Figure 2B). No association was found between RI value and glomerulosclerosis. RI tended to increase with the percentage of interstitial fibrosis (n = 58, r = 0.222, p = 0.10) and was significantly higher when interstitial fibrosis exceeded 20% (0.67 [0.55-1.00] vs 0.61 [0.31- 0.83], p = 0.014) (Figure 2C). Finally, patients with isolated glomerular involvement had significant lower RI than those with interstitial fibrosis > 5% and/or vascular lesions (hyaline arterial deposits and/or intima thickening) (0.60 [0.47-0.64] vs 0.64 [0.31-1.00], p = 0.05).

### Association of renal arterial resistive index with renal function outcome

Renal function outcome was assessed at 18 months in 35 (60%) patients (Table 2). Among them, 13 (37%) patients experienced renal function decline. Seven patients had a decrease in eGFR of at least 5 mL/min/1.73 m^2^/year and 6 needed chronic RRT. Their RI was significantly higher than RI of patients with stable or improved renal function (0.69 [0.63-1.00] vs 0.61 [0.31-0.70], p = 0.0023) (Table 2). In univariate analysis, other factors associated with renal function decline at 18 months were: age (p = 0.0035), baseline eGFR (p = 0.052) and proteinuria/creatininuria ratio (p = 0.049) (Table 2). Baseline RI ≥ 0.65 (p = 0.0075) and age (p = 0.037) were the only independent factors associated with renal function decline at 18 months identified by multiple logistic regression (Table 3).

**Table 2:** **Univariate analysis of parameters associated with renal function decline at 18 months (defined as a decrease in eGFR of at least 5 mL/min/ 1.73 m**^**2**^**/year or need for RRT) (Mann–Whitney and Fisher’s exact tests)**

	**No decline**	**Decline**	**p**
**Number (%) of patients**	**22 (63%)**	**13 (37%)**	
**Age (years)**	**38 (23–68)**	**55 (24–79)**	**0.0035**
**Baseline eGFR (mL/min/1.73m**^**2**^**)**	**65 (17–108)**	**12 (4–65)**	**0.052**
**eGFR(mL/min/1.73m**^**2**^**) at 18 months**	**69 (24–123)**	**8 (5–70)**	**0.000015**
SBP (mmHg)	127 (98–160)	129 (98–163)	0.30
DBP (mmHg)	77 (62–91)	70 (59–96)	0.89
**Proteinuria/creatininuria (mg/mmol)**	**96 (7–1216)**	**492 (69–1742)**	**0.049**
% sclerotic glomeruli	16 (0–71)	4 (0–80)	0.88
% interstitial fibrosis	18 (0–80)	18 (0–90)	0.76
**RI**	**0.61 (0.31-0.70)**	**0.69 (0.63-1.00)**	**0.0023**
**Number (%) of patients with RI ≥ 0.65**	**4 (18%)**	**10 (77%)**	**0.0011**

**Table 3:** **Multivariate analysis of parameters associated with renal function decline at 18 months (defined as a decrease in eGFR of at least 5 mL/min/ 1.73 m**^**2**^**/year or need for RRT) (logistic regression, n=35 patients)**

	**OR**	**95% CI**	**P**
**Proteinuria/creatininuria (mg/mmol)**	**1.001**	**0.998-1.003**	**0.56**
**Baseline eGFR (mL/min/1.73m**^**2**^**)**	**0.977**	**0.936-1.021**	**0.30**
**Age (years)**	**1.079**	**0.996-1.169**	**0.062**
**RI ≥ 0.65**	**7.751**	**1.045-57.479**	**0.045**
	**OR**	**95% CI**	**P**
**Age (years)**	**1.078**	**1.004-1.158**	**0.037**
**RI ≥ 0.65**	**13.04**	**1.984-85.727**	**0.0075**

### Which RI threshold should be used in clinical practice?

**Figure 3:**
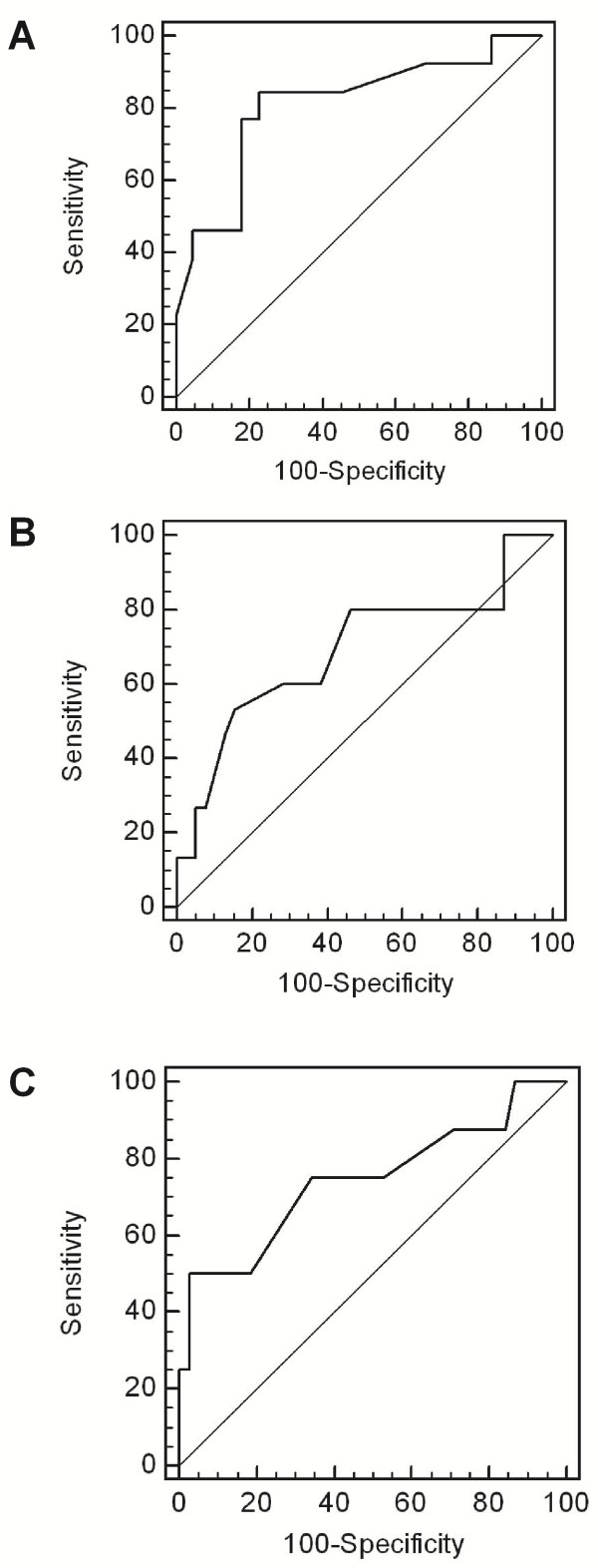
**Receiver operating characteristic (ROC) curves for RI to discriminate.A**: renal function decline at 18 months (AUC = 0.809, p = 0.0002). **B**: interstitial fibrosis > 20% (AUC = 0.690, p = 0.037). **C**: severe arteriosclerosis (AUC = 0.740, p = 0.039).

**Figure 4:**
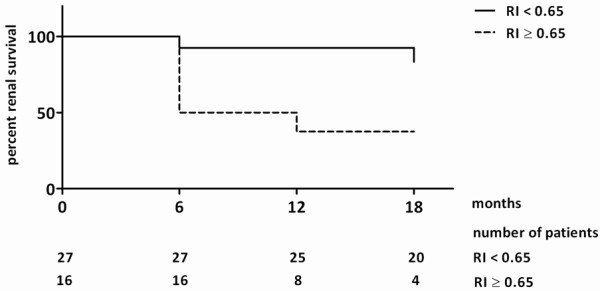
**Kaplan Meier curves of renal survival according to baseline RI.** Renal function decline is defined as decrease in eGFR of at least 5mL/min/1.73m^2^/year or need for RRT. Plain line represents patients with RI < 0.65 and dotted line those with RI ≥ 0.65; p = 0.0005, log-rank test.

In order to define the most accurate threshold, we draw receiver operating characteristic (ROC) curves (Figure 3). According to the ROC curves analysis, 0.65 threshold was the most discriminant for renal function decline at 18 months, interstitial fibrosis exceeding 20% and severe arteriosclerosis. Analysis of renal survival using Kaplan-Meier curves confirmed that patients with baseline RI ≥ 0.65 had a poorer renal outcome than those with baseline RI < 0.65 (p = 0.0005, Log-Rank test) (Figure 4). Sensitivity, specificity, predictive positive and predictive negative values of RI ≥ 0.65 for renal function decline at 18 months were respectively 77%, 86%, 71% and 82%.

## Discussion

The present study shows that initial measurement of RI in patients with various nephropathies at time of renal biopsy is clinically relevant for several reasons. We show that RI is associated with renal function and pulse pressure, a surrogate marker of arterial stiffness. More importantly, RI is associated with severe interstitial fibrosis and arteriosclerosis and eGFR decline. Previous studies reported either the association of RI with interstitial fibrosis, tubulo-interstitial lesions [9-12,25], or vascular lesions [9-11] or renal outcome in CKD [9,11,13,15,16]. To our knowledge, none of these studies simultaneously evaluated the association of RI with the main chronic renal histological lesions and with renal function outcome. Furthermore, the most relevant threshold of RI for clinical practice was still debated. Here, the cut-off value of 0.65 was the most discriminant for severe arteriosclerosis, extended fibrosis and renal function decline.

In this study, we analyzed the association of RI with pathological changes and renal function outcome in an unselected population of 58 patients with various nephropathies and renal function alterations. In order to be closer to clinical practice, our inclusion criteria differ from other studies which were done in specific renal diseases [12,25]. Our results emphasize the general predictive value of RI in CKD patients independently of the type of nephropathy. Secondly, only well-defined chronic renal lesions as interstitial fibrosis, percentage of sclerotic glomeruli, arteriosclerosis lesions were taken into account in our study. These criteria differ from previous studies which used combined scores as tubulo-interstitial injury or interstitial fibrosis/tubular atrophy, which could reflect acute kidney injury [9-12]. Despite a relative small population of CKD patients, we found a significant association of RI with severe renal lesions and renal function decline, consistently with previous studies [9-16,20].

As previous authors, we found a positive correlation between RI and age [10,26-29] and in a lesser extent with pulse pressure [26,30-33]. Prior studies reported an association between RI and other markers of arterial stiffness as pulse wave velocity [31,34] and ankle-brachial blood pressure index [35]. In our study, RI was not associated with systolic nor with diastolic blood pressure despite its relationship with pulse pressure. This suggests that the elevation of RI rather reflects the vascular consequences of hypertension than hypertension itself. An alternative explanation is the lack of power of our study. Nevertheless, this result was demonstrated by previous authors who showed that RI is a marker of target organ damage in essential hypertension [30,32,34,36,37] as left ventricular hypertrophy, carotid and coronary atherosclerosis. Other studies demonstrated that high RI was also associated with systemic atherosclerosis in diabetic patients [31,38] and renal transplant recipients [35]. Moreover, Pearce *et al.* recently showed that elevated renal RI is predictive of cardiovascular events in the elderly [39].

The elevation of RI with severe arteriosclerosis may account for its association with cardiovascular risk. As previous authors [9,25], we actually found an association between RI and renal arteriosclerosis. It is interesting to note that only patients with severe intima thickening exhibited high RI. Nor hyaline arteriolar deposits nor moderate intima thickening were associated with increased RI. This could be due to the lack of power of our study. Nevertheless, it could also suggest that the presence of moderate vascular lesions is not sufficient to induce an elevation of RI and that these alterations must be important enough to reduce artery lumen, raise arterial stiffness and vascular resistance, and consequently generate an increase in RI. Overall, these findings suggest that high RI reflects severe renal arteriosclerosis and maybe systemic arteriosclerosis.

Our study also questioned the correlation of RI with renal fibrosis. We did not find any relationship between RI and glomerulosclerosis. Moreover, RI was significantly lower in case of isolated glomerular involvement, i.e. without any vascular and/or tubulo-interstitial damage. This result corroborates previous findings of Platt *et al.*[25]. Only one among three previous studies found a significant association between glomerulosclerosis and RI [10]. This correlation was weak in a second study [12] and not significant in a multivariate analysis in the third one [9]. Altogether, these findings suggest that glomerulosclerosis does not influence the value of RI.

On the other hand, as other authors, we found an association between RI value and the extension of interstitial fibrosis [9,11,12,25] and the severity of renal impairment [13,26,40-44]. Median percentage of interstitial fibrosis was six fold higher in patients with RI exceeding 0.65. As arteriosclerosis, interstitial fibrosis appears as an important determinant of RI. Three hypotheses can be drawn about the physiopathological mechanisms involved in elevation of RI with the progression of CKD: i) decrease in arterial compliance and increase in vascular resistance because of renal arteriosclerosis, ii) elevation of pressure exerted by interstitial fibrosis on adjacent vessels, iii) vasoconstriction secondary to the hypoxia induced by the previous phenomena and by the loss of capillaries associated with renal fibrosis. These mechanisms are probably combined and our results do not allow us to precise which one contributes the most to the elevation of RI.

In the second part of our study, we found that high RI was independently associated with accelerated renal function decline. Sugiura *et al.*[13] reported similar result in a larger cohort of 311 CKD patients followed-up for two years. Such results were also found by other authors in CKD [11,14-17], essential hypertension [17] and renal transplantation [8,45]. Our results extend previous findings to a population of patients with various nephropathies. The concomitant association of RI with interstitial fibrosis and arteriosclerosis which are known to be major determinants of the progression of CKD [4] may explain its prognosis value.

Finally, we attempted to define the most relevant threshold of RI in clinical practice. Consistently with the first findings of Sugiura *et al*. [12], we found that 0.65 was the most accurate threshold to detect extent interstitial fibrosis, but also severe arteriosclerosis. Nevertheless, more recently, Sugiura *et al*. suggested that 0.70 threshold was better than 0.65 cut-off to predict renal function decline [13]. In contrast, we found by ROC curves analysis that RI ≥ 0.65 has the best sensitivity (77%) and specificity (86%) to discriminate renal function decline. This discrepancy may be explained by the different definitions of renal function decline used in the two studies. Using the criteria of at least 10 mL/min/ 1.73 m^2^/year, Sugiura *et al*. may have selected more severe patients. In cohort studies, mean slope of eGFR decline is less than 5 mL/min/ 1.73 m^2^ in most CKD patients [2,19-23]. A decrease of 5 mL/min/ 1.73 m^2^/year appears to be closer to clinical practice and more helpful to detect a larger of number patients at high risk of accelerated CKD progression.

Our study has several limits. We mostly included glomerulonephritis and few vascular and tubulo-interstitial diseases. Because of the weak proportion of chronic vascular and tubulo-interstitial diseases, we can wonder whether our results could apply to those nephropathies. Nevertheless, several previous studies found an association between high RI and poor renal outcome in essential hypertension and chronic tubulo-interstitial nephropathies [13,14,17,34,37]. The main limit of our study is the non exhaustive collection of renal function data. However, our results corroborate those of several previous studies [9,11,13,15,16].

## Conclusion

Our results show that RI ≥ 0.65 in CKD patients with various nephropathies is associated with extended interstitial fibrosis, severe arteriosclerosis and renal function decline. Consistently with previous findings, these results suggest that non invasive US Doppler measurement of RI could give the opportunity to identify CKD patients at high risk of ESRD and help clinicians in their management. Our results need to be confirmed in a larger targeted intervention multicentric study of outpatients.

## Abbreviations

CKD: Chronic kidney disease; DBP: Diastolic blood pressure; eGFR: Estimated glomerular filtration rate; ESRD: End stage renal disease; FSGN: Focal segmental glomerulonephritis; HIVAN: HIV-associated nephropathy; RI: Renal doppler resistive index; RRT: Renal replacement therapy; SBP: Systolic blood pressure.

## Competing interests

None of the authors have conflicts of interest to declare.

## Authors’ contributions

JJB designed the study. NB and JJB had full access to all the data and take responsibility for the integrity of the data and the accuracy of the data analysis. NB and VJ collected data. PC realized histological study. VC and JMF performed renal US Doppler. NB and PL realized statistical analysis. NB and JJB wrote the manuscript. PL and PR made critical revision of the manuscript. All authors approved the final version of this manuscript.

## Pre-publication history

The pre-publication history for this paper can be accessed here:

http://www.biomedcentral.com/1471-2369/13/139/prepub

## References

[B1] Meguid El Nahas A Bello AK Chronic kidney disease: the global challenge Lancet 2005 365 9456 331 340 15664230 10.1016/S0140-6736(05)17789-7

[B2] Eriksen BO Ingebretsen OC The progression of chronic kidney disease: a 10-year population-based study of the effects of gender and age Kidney Int 2006 69 2 375 382 10.1038/sj.ki.5000058 16408129

[B3] Clark LE Khan I Outcomes in CKD: what we know and what we need to know Nephron 2010 114 2 c95 c102 19887829 10.1159/000254381

[B4] Nath KA Tubulointerstitial changes as a major determinant in the progression of renal damage Am J Kidney Dis 1992 20 1 1 17 1621674 10.1016/s0272-6386(12)80312-x

[B5] Crutchley TA Pearce JD Craven TE Stafford JM Edwards MS Hansen KJ Clinical utility of the resistive index in atherosclerotic renovascular disease J Vasc Surg 2009 49 1 148 155 155 e141-143; discussion 155 10.1016/j.jvs.2008.08.008 18951751

[B6] Radermacher J Chavan A Bleck J Vitzthum A Stoess B Gebel MJ Galanski M Koch KM Haller H Use of Doppler ultrasonography to predict the outcome of therapy for renal-artery stenosis N Engl J Med 2001 344 6 410 417 10.1056/NEJM200102083440603 11172177

[B7] Rodrigo E Lopez-Rasines G Ruiz JC Lastra P Gomez-Dermitt V Gomez-Alamillo C Gonzalez-Cotorruelo J Calabia A Arias M Determinants of resistive index shortly after transplantation: independent relationship with delayed graft function Nephron 2010 114 3 c178 c186 19955823 10.1159/000262300

[B8] Radermacher J Mengel M Ellis S Stuht S Hiss M Schwarz A Eisenberger U Burg M Luft FC Gwinner W The renal arterial resistance index and renal allograft survival N Engl J Med 2003 349 2 115 124 10.1056/NEJMoa022602 12853584

[B9] Ikee R Kobayashi S Hemmi N Imakiire T Kikuchi Y Moriya H Suzuki S Miura S Correlation between the resistive index by Doppler ultrasound and kidney function and histology Am J Kidney Dis 2005 46 4 603 609 10.1053/j.ajkd.2005.06.006 16183414

[B10] Mostbeck GH Kain R Mallek R Derfler K Walter R Havelec L Tscholakoff D Duplex Doppler sonography in renal parenchymal disease. Histopathologic correlation J Ultrasound Med 1991 10 4 189 194 2051529 10.7863/jum.1991.10.4.189

[B11] Splendiani G Parolini C Fortunato L Sturniolo A Costanzi S Resistive index in chronic nephropathies: predictive value of renal outcome Clin Nephrol 2002 57 1 45 50 11837800 10.5414/cnp57045

[B12] Sugiura T Nakamori A Wada A Fukuhara Y Evaluation of tubulointerstitial injury by Doppler ultrasonography in glomerular diseases Clin Nephrol 2004 61 2 119 126 14989631 10.5414/cnp61119

[B13] Sugiura T Wada A Resistive index predicts renal prognosis in chronic kidney disease Nephrol Dial Transplant 2009 24 9 2780 2785 10.1093/ndt/gfp121 19318356

[B14] Parolini C Noce A Staffolani E Giarrizzo GF Costanzi S Splendiani G Renal resistive index and long-term outcome in chronic nephropathies Radiology 2009 252 3 888 896 10.1148/radiol.2523080351 19528356

[B15] Petersen LJ Petersen JR Talleruphuus U Ladefoged SD Mehlsen J Jensen HA The pulsatility index and the resistive index in renal arteries. Associations with long-term progression in chronic renal failure Nephrol Dial Transplant 1997 12 7 1376 1380 10.1093/ndt/12.7.1376 9249772

[B16] Radermacher J Ellis S Haller H Renal resistance index and progression of renal disease Hypertension 2002 39 2 Pt 2 699 703 11882634 10.1161/hy0202.103782

[B17] Okura T Kurata M Irita J Enomoto D Jotoku M Nagao T Koresawa M Kojima S Hamano Y Mashiba S Renal resistance index is a marker of future renal dysfunction in patients with essential hypertension J Nephrol 2010 23 2 175 180 20119927

[B18] National Kidney Foundation K/DOQI clinical practice guidelines for chronic kidney disease: evaluation, classification, and stratification Am J Kidney Dis 2002 39 2 Suppl 1 S1 S266 11904577

[B19] Conway B Webster A Ramsay G Morgan N Neary J Whitworth C Harty J Predicting mortality and uptake of renal replacement therapy in patients with stage 4 chronic kidney disease Nephrol Dial Transplant 2009 24 6 1930 1937 10.1093/ndt/gfn772 19181760

[B20] Eriksen BO Ingebretsen OC In chronic kidney disease staging the use of the chronicity criterion affects prognosis and the rate of progression Kidney Int 2007 72 10 1242 1248 10.1038/sj.ki.5002472 17687256

[B21] Halbesma N Kuiken DS Brantsma AH Bakker SJ Wetzels JF De Zeeuw D De Jong PE Gansevoort RT Macroalbuminuria is a better risk marker than low estimated GFR to identify individuals at risk for accelerated GFR loss in population screening J Am Soc Nephrol 2006 17 9 2582 2590 10.1681/ASN.2005121352 16899519

[B22] Hunsicker LG Adler S Caggiula A England BK Greene T Kusek JW Rogers NL Teschan PE Predictors of the progression of renal disease in the modification of diet in renal disease study Kidney Int 1997 51 6 1908 1919 10.1038/ki.1997.260 9186882

[B23] John R Webb M Young A Stevens PE Unreferred chronic kidney disease: a longitudinal study Am J Kidney Dis 2004 43 5 825 835 10.1053/j.ajkd.2003.12.046 15112173

[B24] Farris AB Adams CD Brousaides N Della Pelle PA Collins AB Moradi E Smith RN Grimm PC Colvin RB Morphometric and visual evaluation of fibrosis in renal biopsies J Am Soc Nephrol 2011 22 1 176 186 10.1681/ASN.2009091005 21115619 PMC3014046

[B25] Platt JF Ellis JH Rubin JM DiPietro MA Sedman AB Intrarenal arterial Doppler sonography in patients with nonobstructive renal disease: correlation of resistive index with biopsy findings Ajr 1990 154 6 1223 1227 2110732 10.2214/ajr.154.6.2110732

[B26] Heine GH Gerhart MK Ulrich C Kohler H Girndt M Do ultrasound renal resistance indices reflect systemic rather than renal vascular damage in chronic kidney disease? Nephrol Dial Transplant 2006 22 1 163 170 10.1093/ndt/gfl484 16936334

[B27] Keogan MT Kliewer MA Hertzberg BS DeLong DM Tupler RH Carroll BA Renal resistive indexes: variability in Doppler US measurement in a healthy population Radiology 1996 199 1 165 169 8633141 10.1148/radiology.199.1.8633141

[B28] Lin ZY Wang LY Yu ML Dai CY Chen SC Chuang WL Hsieh MY Tsai JF Chang WY Influence of age on intrarenal resistive index measurement in normal subjects Abdom Imaging 2003 28 2 230 232 10.1007/s00261-002-0024-4 12592470

[B29] Rivolta R Cardinale L Lovaria A Di Palo FQ Variability of renal echo-Doppler measurements in healthy adults J Nephrol 2000 13 2 110 115 10858972

[B30] Florczak E Januszewicz M Januszewicz A Prejbisz A Kaczmarska M Michalowska I Kabat M Rywik T Rynkun D Zielinski T Relationship between renal resistive index and early target organ damage in patients with never-treated essential hypertension Blood Press 2009 18 1–2 55 61 19353412 10.1080/08037050902864078

[B31] Ohta Y Fujii K Arima H Matsumura K Tsuchihashi T Tokumoto M Tsuruya K Kanai H Iwase M Hirakata H Increased renal resistive index in atherosclerosis and diabetic nephropathy assessed by Doppler sonography J Hypertens 2005 23 10 1905 1911 10.1097/01.hjh.0000181323.44162.01 16148615

[B32] Okura T Watanabe S Miyoshi K Fukuoka T Higaki J Intrarenal and carotid hemodynamics in patients with essential hypertension Am J Hypertens 2004 17 3 240 244 10.1016/j.amjhyper.2003.10.005 15001198

[B33] Ozelsancak R Torun D Koc Z Sezer S Ozdemir FN Niron EA Relationship between renal resistive index and inflammation in untreated hypertensive patients Int Heart J 2009 50 6 753 761 10.1536/ihj.50.753 19952472

[B34] Raff U Schmidt BM Schwab J Schwarz TK Achenbach S Bar I Schmieder RE Renal resistive index in addition to low-grade albuminuria complements screening for target organ damage in therapy-resistant hypertension J Hypertens 2010 28 3 608 614 10.1097/HJH.0b013e32833487b8 20090556

[B35] Heine GH Gerhart MK Ulrich C Kohler H Girndt M Renal Doppler resistance indices are associated with systemic atherosclerosis in kidney transplant recipients Kidney Int 2005 68 2 878 885 10.1111/j.1523-1755.2005.00470.x 16014069

[B36] Pontremoli R Viazzi F Martinoli C Ravera M Nicolella C Berruti V Leoncini G Ruello N Zagami P Bezante GP Increased renal resistive index in patients with essential hypertension: a marker of target organ damage Nephrol Dial Transplant 1999 14 2 360 365 10.1093/ndt/14.2.360 10069189

[B37] Shimizu Y Itoh T Hougaku H Nagai Y Hashimoto H Sakaguchi M Handa N Kitagawa K Matsumoto M Hori M Clinical usefulness of duplex ultrasonography for the assessment of renal arteriosclerosis in essential hypertensive patients Hypertens Res 2001 24 1 13 17 10.1291/hypres.24.13 11213024

[B38] Buscemi S Verga S Batsis JA Cottone S Mattina A Re A Arnone M Citarda S Cerasola G Intra-renal hemodynamics and carotid intima-media thickness in the metabolic syndrome Diabetes Res Clin Pract 2009 86 3 177 185 10.1016/j.diabres.2009.09.015 19815301

[B39] Pearce JD Craven TE Edwards MS Corriere MA Crutchley TA Fleming SH Hansen KJ Associations between renal duplex parameters and adverse cardiovascular events in the elderly: a prospective cohort study Am J Kidney Dis 2010 55 2 281 290 10.1053/j.ajkd.2009.10.044 20116688 PMC2933103

[B40] Galesic K Sabljar-Matovinovic M Tomic M Brkljacic B Renal vascular resistance in glomerular diseases–correlation of resistance index with biopsy findings Coll Antropol 2004 28 2 667 674 15666598

[B41] Kim SH Kim WH Choi BI Kim CW Duplex Doppler US in patients with medical renal disease: resistive index vs serum creatinine level Clin Radiol 1992 45 2 85 87 10.1016/S0009-9260(05)80060-1 1737434

[B42] Petersen LJ Petersen JR Ladefoged SD Mehlsen J Jensen HA The pulsatility index and the resistive index in renal arteries in patients with hypertension and chronic renal failure Nephrol Dial Transplant 1995 10 11 2060 2064 8643168

[B43] Yura T Yuasa S Sumikura T Takahashi N Aono M Kunimune Y Fujioka H Miki S Takamitsu Y Matsuo H Doppler sonographic measurement of phasic renal artery blood flow velocity in patients with chronic glomerulonephritis J Ultrasound Med 1993 12 4 215 219 8497028 10.7863/jum.1993.12.4.215

[B44] Sari A Dinc H Zibandeh A Telatar M Gumele HR Value of resistive index in patients with clinical diabetic nephropathy Invest Radiol 1999 34 11 718 721 10.1097/00004424-199911000-00008 10548384

[B45] Saracino A Santarsia G Latorraca A Gaudiano V Early assessment of renal resistance index after kidney transplant can help predict long-term renal function Nephrol Dial Transplant 2006 21 10 2916 2920 10.1093/ndt/gfl203 16891640

